# Impact of Air Pollution Control Devices on VOC Profiles and Emissions from Municipal Waste Incineration Plant

**DOI:** 10.3390/toxics13121067

**Published:** 2025-12-11

**Authors:** Jun Liu, Duanhe Zhao, Fei Wu, Huanhuan Luo, Daxiang Hou, Yue Peng

**Affiliations:** 1 School of Petroleum and Natural Gas Engineering, Changzhou University, Changzhou 213164, China; s24040858026@smail.cczu.edu.cn (D.Z.); s24040858029@smail.cczu.edu.cn (D.H.); 2 Key Laboratory of Oil-Gas & New-Energy Storage and Transportation Technology of Jiangsu Province Higher Education Institutes, Changzhou University, Changzhou 213164, China; pengyue@bipt.edu.cn; 3 Huaneng Hunan Yueyang Power Generation Co., Ltd., Yueyang 414000, China; wufei1503@163.com (F.W.); lh10172025@163.com (H.L.); 4 Department of Environmental Engineering, Beijing Institute of Petrochemical Technology, Qingyuan North Road 19, Beijing 102617, China

**Keywords:** municipal waste incineration (MWI), volatile organic compounds (VOCs), air pollution control devices (APCDs), emission, synergistic removal

## Abstract

With the rapid development of urbanization, municipal waste incineration (MWI) has become the primary method of waste disposal in urban areas, leading to growing concerns about volatile organic compounds (VOC) emissions. This study conducted full-process VOC field sampling at a representative MWI plant in China to investigate the emission characteristics and removal efficiencies of air pollution control devices (APCDs). A total of 59 VOC species were identified in the flue gas, including 5 alkanes/alkenes, 14 aromatics, 8 oxygenated-VOCs, and 32 halogenated hydrocarbons. The activated carbon injection combined with fabric filters and wet desulfurization tower demonstrated varying removal efficiencies across VOC groups, with synergistic removal efficiencies being ranked as follows: alkanes/alkenes (90.9%) > aromatics (87.0%) > halogenated hydrocarbons (61.3%) > O-VOCs (42.2%). The total VOC removal efficiency reached 77.5%. The VOCs emission factor of the MWI plant was calculated as (1.9 ± 0.6) × 10^3^ ng/g-waste, which would rise to (8.4 ± 2.1) × 10^3^ ng/g-waste in the absence of APCDs. This indicates that the current APCD system reduces VOC emissions by approximately 6.52 × 10^4^ g annually from this MWI plant, highlighting the crucial role of multistage APCDs in mitigating VOC pollution.

## 1. Introduction

With the rapid pace of urbanization and rising living standards in China, municipal waste generation has continued to grow steadily. By the end of 2023, the total amount of municipal waste collected and transported in China had reached 254 million tons [1]. Among the mainstream waste treatment methods (such as landfilling, incineration, and composting) [2], waste incineration has become the dominant approach in urban areas, primarily due to its exceptional capacity for volume reduction, effective land use, and potential for energy recovery [3]. Over the past decade, the number of municipal waste incineration (MWI) plants in China has increased from 188 to 696, and the proportion of waste treated by incineration has risen from 29.8% to 82.5% [1].

However, the environmental impacts of MWI plants have drawn increasing attention. Traditionally, the primary environmental concerns associated with incineration have centered on polychlorinated dibenzo-p-dioxins and dibenzofurans (PCDD/Fs) and toxic heavy metals [4]. This prioritization is well-founded, given their extreme toxicity, environmental persistence, and bioaccumulative potential, which have necessitated the implementation of rigorous emission standards and advanced abatement technologies. Yet, as the emissions of these conventional pollutants are increasingly well-controlled by mature air pollution control device (APCD) systems, the significance of volatile organic compounds (VOCs) has emerged as a critical but often overlooked issue. These pollutants not only pose direct threats to human health, with certain VOCs identified as carcinogenic, teratogenic, and mutagenic agents [5,6], but also serve as crucial precursors for ozone and secondary organic aerosols [7], thereby contributing significantly to regional photochemical smog and haze formation. Consequently, shifting the research focus to the characterization and control of VOCs from MWI plants has become an urgent priority for further improving air quality.

Previous research on VOCs emitted from MWI plants has mainly focused on sampling at the stack outlet to determine VOC species and concentrations. Studies conducted in Switzerland, South China, Central China, and the North China Plain have reported notable differences in VOC composition and levels, with total concentrations ranging from 16.75 μg/m^3^ to 4.28 mg/m^3^ [8,9,10,11]. These variations are generally attributed to differences in waste composition, sampling approaches, and, most notably, the configurations and operating conditions of APCD systems of MWI plants. Although these studies have greatly advanced the understanding of VOCs emitted from MWI plants, they provide very limited insight into how individual APCD units and their combinations affect the distribution of VOCs throughout the entire flue gas purification process.

To investigate the removal performance of APCDs, several studies have examined VOCs’ behavior in coal-fired power plants [12,13,14,15]. Cheng et al. [12] evaluated VOC concentrations at the inlets and outlets of selective catalytic reduction (SCR), electrostatic precipitator (ESP), flue gas desulfurization (FGD), and wet electrostatic precipitator (WESP) units, reporting corresponding removal efficiencies of 64.4%, –12.5%, 16.1%, and 8.7%. These changes were attributed to oxidation, deposition, condensation, and water absorption mechanisms. Similarly, Ge et al. [13] assessed the synergistic removal of VOCs by the SCR–APH–ESP–FGD system in a 330 MW coal-fired unit. VOC concentrations decreased from 1.084 mg/m^3^ to 0.593 mg/m^3^, 0.049 mg/m^3^, 0.108 mg/m^3^, and 0.088 mg/m^3^ across the respective units, with removal efficiencies of 45%, 92%, −122%, and 18%. Their analysis further showed that high-voltage discharges in ESPs could induce desorption of VOCs from fly ash, leading to an unexpected increase in VOC concentrations at the ESP outlet. However, these studies were conducted in coal-fired power plants, whose fuel characteristics, flue-gas composition, and APCD configurations differ fundamentally from those of MWI plants. Therefore, the findings obtained from coal-fired power plants cannot be directly generalized to MWI plants operations. Furthermore, a comprehensive full-process investigation encompassing all major APCD stages in MWI plants has not yet been reported, leaving the synergistic removal behavior and mechanisms of volatile organic compounds in incineration power plant APCD systems largely unresolved.

In this study, a comprehensive multi-point sampling of volatile organic compounds (VOCs) was conducted across the entire flue gas treatment process of a representative full-scale MWI plant in China, utilizing a modified adsorbent tube sampling system designed to withstand high particulate matter and humidity conditions. By examining the variations in VOC species and concentrations across key APCD units, including the activated carbon injection combined with fabric filter (ACI + FF) system and the wet desulfurization tower (WDT). This study aims to: (1) quantify the removal contributions of individual and combined APCD units, (2) elucidate the mechanisms governing VOCs abatement in ACI + FF and WDT processes, and (3) determine the VOC emission factor for the MWI plant and estimate the potential annual emission reductions achieved by the APCD system. Although based on a single facility, the mechanistic insights derived herein are broadly applicable to other MWI plants, with consideration of local waste characteristics and operating conditions. The findings provide a scientific basis for the precise control and optimized management of VOC emissions in the waste incineration sector.

## 2. Materials and Methods

### 2.1. Municipal Waste

In this study, pretreated municipal waste was randomly collected from the MWI plant. After sterilization and drying, the mixed wastes were sorted into specific categories and quantified by mass. The distribution of these categories is listed in Table 1. Overall, the materials were dominated by organics, accounting for 87% of the total mass. These organic fractions were composed mainly of plastic/rubber products, cotton fabric, woodware, food waste, medicine, and paper products.

To further characterize the feedstock, the organic portions of the waste were shredded into ~0.5 mm fragments following drying. Ultimate analysis of carbon, hydrogen, nitrogen, and sulfur was performed using an elemental analyzer (EA3000, EuroVector, Pavia, Italy). Proximate analysis, including moisture, volatile matter, and ash, was conducted with a thermogravimetric analyzer (TGA701, LECO, St Joseph, MI, USA) following ASTM D7582 [16]. The higher heating value was determined via a bomb calorimeter (VBR-6000/D, Volband, Changsha, China) according to ASTM D5865-04 [17]. A summary of the major physicochemical properties of the representative municipal waste materials is presented in Table 2.

### 2.2. Overview of Waste Incineration Plants

**Figure 1:**
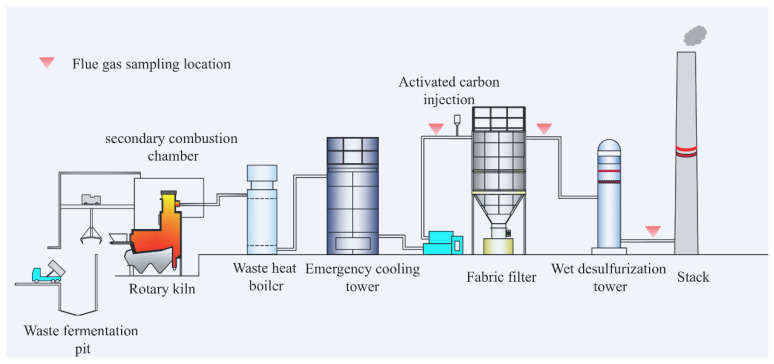
Schematic drawing of the MWI plant and flue gas sampling locations. Created by the authors.

The waste incineration technological process of the MWI plant is illustrated in Figure 1. The facility has a designed annual capacity of approximately 10,000 tons and operates for about 8000 h per year. It employs a rotary kiln incineration system consisting of a feeder, rotary kiln, secondary combustion chamber, waste-heat boiler, emergency quench tower, ACI unit, FF, induced draft fan, and a WDT device.

Before incineration, municipal waste undergoes short-term fermentation in the temporary storage pit and is subsequently fed into the rotary kiln. Complete combustion is ensured in the secondary combustion chamber. The flue gas is then rapidly cooled to below 200 °C by the waste-heat boiler followed by the emergency quench tower to inhibit de novo dioxin formation. After cooling, the gas stream passes sequentially through a series of APCD systems.

The first unit is the ACI system, where powdered activated carbon is introduced to adsorb dioxins, ensuring that dioxin emissions remain below 0.1 ng TEQ/m^3^. The flue gas then enters the FF device, which removes particulate matter together with the activated carbon particles loaded with adsorbed pollutants, effectively reducing PM emissions to below 5 mg/m^3^. Finally, the gas flows into the WDT device, where acidic gases such as SO_2_, HCl, and NOx are removed through liquid–gas absorption using Ca(OH)_2_. The corresponding emission concentrations of NOx, SO_2_, and HCl are controlled to below 100 mg/m^3^, 5 mg/m^3^, and 1 mg/m^3^, respectively. The temperatures at the inlets and outlets of each APCD unit are also provided in Figure 1.

### 2.3. Field Sampling Method

The sampling of VOCs in the incineration flue gas was conducted in accordance with the standard method HJ 734—2014 [18], using adsorption tubes filled with adsorbents (activated carbon, Tenax^®^ GC, XAD-2, Charlotte, NC, USA) to collect VOCs from the flue gas. Each sampling unit consisted of two sorbent tubes connected in series. The second tube served exclusively as a breakthrough check, ensuring that VOCs did not penetrate the first tube during sampling. To improve sampling accuracy, slight modifications were made to the sampling system as follows: (1) The system utilized a sampling probe compliant with EPA Method 17, equipped with a pre-filter cartridge designed to capture particulate matter in the flue gas and prevent interference with VOC collection; (2) a Nafion dryer was installed upstream of the sorbent tube to remove moisture and minimize its impact on VOC adsorption efficiency; (3) an oxygen sensor was incorporated to continuously monitor the integrity and airtightness of the sampling system in real time. During sampling, the entire system was maintained at a constant temperature of 120 °C to ensure that VOCs did not condense during transmission. To ensure the reliability of the sampling results, three parallel samples were collected at each sampling point, with each sampling event lasting approximately 40 min (20 L of flue gas at a flow rate of 0.5 L/min). All sampling was completed within two consecutive days under stable operating conditions, allowing the influence of fuel composition fluctuations to be reasonably considered negligible. Sampling points were located at three key positions along the process: before the BACI + FF system (BACI + FF, 172 °C), after the BACI + FF system (AACI + FF, 156 °C), and after the wet desulfurization tower device (AWDT, 73 °C), as marked by red dots in Figure 1. Upon completion of sampling, both ends of the adsorption tubes were immediately sealed with polytetrafluoroethylene (PTFE) caps and stored at 4 °C in a dark environment. All samples were analyzed within 7 days to ensure data reliability.

### 2.4. Analytical Method

The analysis of the adsorption tube samples was carried out using thermal desorption (TD; BCHP, JX-5, Beijing, China) coupled with gas chromatography–mass spectrometry (GC-MS; PerkinElmer, Clarus SQ 8 T, Waltham, MA, USA). During analysis, the adsorption tubes were purged with high-purity helium gas (≥99.999%) at a flow rate of 30 mL/min for 3 min at 300 °C to release VOCs. The desorbed compounds were trapped by a −30 °C graphitized carbon cold trap and subsequently flash-heated to 300 °C for injection into the GC-MS system. Chromatographic separation was performed using a DB-5 capillary column (60 m × 0.25 mm × 0.25 μm), with helium as the carrier gas at a flow rate of 1.0 mL/min. The temperature program was as follows: initial temperature of 38 °C held for 5 min, ramped at 10 °C/min to 280 °C, and held for another 5 min. The mass spectrometer was operated with an ion source temperature of 220 °C and a mass scan range of 35–500 m/z. Quantitative analysis was conducted using an external standard method. Calibration curves (R^2^ ≥ 0.99) were established with standard solutions at concentrations ranging from 10 to 100 μg/mL. Three parallel samples were analyzed at each sampling point (with relative deviation < 15%) to ensure data accuracy. Each batch of samples included blank adsorption tubes and standard quality control samples for quality assurance.

To analyze the VOC emission characteristics of the MWI plant, this study calculated the emission factors (*EFs*) using the following equation:

(1) $$EFs\;=\;C\;\times \;\frac{V}{M}$$

where *EFs* is the VOC emission factor based on the amount of waste incinerated (ng/g-waste), *C* is the concentration of total VOCs (TVOCs) in the flue gas (μg/m^3^), *V* is the hourly flue gas volume (m^3^/h), and *M* is the hourly waste consumption (kg/h).

### 2.5. Quality Control and Quality Assurance

Strict quality control measures were implemented to ensure the accuracy and reliability of experimental data. Prior to sampling, all adsorption tubes were subjected to blank level testing and thermal desorption efficiency evaluation, with acceptable recovery rates for target compounds ranging from 91.5% to 128%. For each sampling batch, at least one full-process blank sample was included. If the absolute mass of any target compound detected in the blank and the second adsorption tube of sampling unit exceeded 7 ng, the entire batch of data was re-evaluated to rule out contamination. During sampling, the flue gas flow rate was maintained within ±10%; if deviations exceeded this range, resampling was required. During analysis, a reference standard solution was injected at the start of each day to check system performance. If the instrument response deviated from the initial calibration curve by more than ±15%, recalibration was immediately conducted.

## 3. Results and Discussion

### 3.1. Characteristics of VOCs Generation During Waste Incineration

In this study, 59 VOCs species were determined in the flue gas at the BACI + FF location, with a total concentration of 1502.6 ± 374.2 μg/m^3^. Based on their chemical composition, the determined VOCs at the BACI + FF location included 5 alkanes/alkenes (accounting for 41.1%), 14 aromatics (20.2%), 8 O-VOCs (6.0%), and 32 halogenated hydrocarbons (32.7%). The names, retention times, and characteristic fragment information of the determined VOCs are summarized in Table S1.

**Figure 2:**
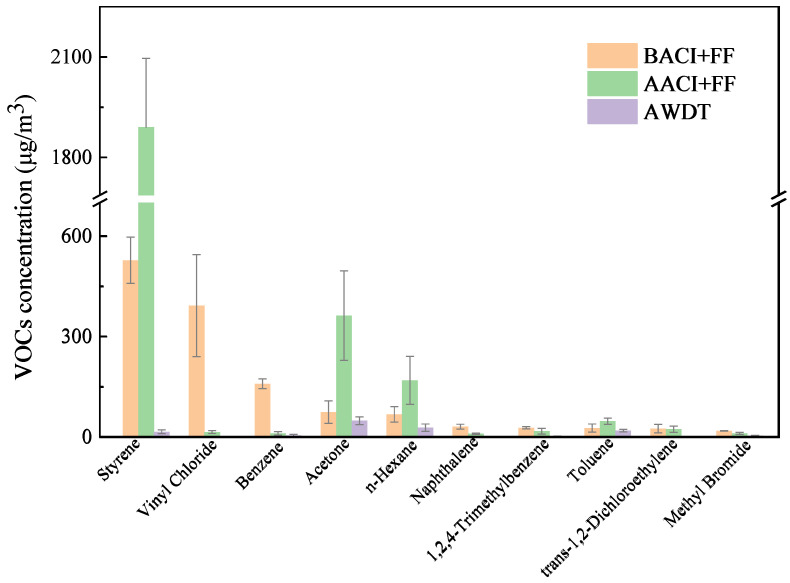
Distribution of the top 10 VOCs by concentration in the MSWI plant.

Figure 2 illustrates the distribution characteristics of the top 10 VOCs by concentration in the flue gas. Styrene ranked first with a concentration of 528.4 ± 69.2 μg/m^3^, accounting for 35.16% of the TVOCs. This phenomenon is primarily attributed to the combustion of materials containing large amounts of waste plastic packaging and paint residues [19]. The next highest concentrations were vinyl chloride (393.1 ± 152.3 μg/m^3^, 26.2%), benzene (160.0 ± 14.2 μg/m^3^, 10.7%), acetone (75.5 ± 33.5 μg/m^3^, 5.0%), n-hexane (68.8 ± 22.9 μg/m^3^, 4.6%), naphthalene (32.0 ± 7.4 μg/m^3^, 2.1%), 1,2,4-trimethylbenzene (28.7 ± 3.3 μg/m^3^, 1.9%), toluene (28.1 ± 12.0 μg/m^3^, 1.9%), trans-1,2-dichloroethylene (26.3 ± 12.3 μg/m^3^, 1.8%), and bromomethane (19.6 ± 0.6 μg/m^3^, 1.3%).

It is noteworthy that the concentration changes in different types of VOCs showed significant variations after treatment by the ACI + FF system and WDT device. To further analyze the removal mechanisms of VOCs by the existing pollution control devices, this study will subsequently investigate the concentration distribution characteristics of the four groups of VOCs.

### 3.2. Distribution Characteristics of Alkanes/Alkenes

**Figure 3:**
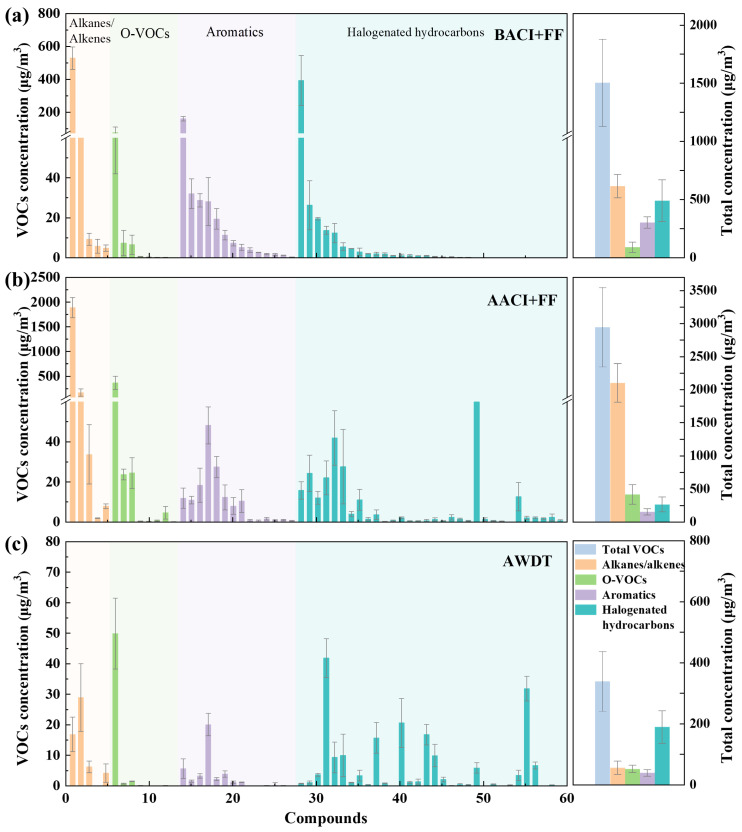
The concentration of VOCs in the flue gas at the sampling location of the (**a**) BACI + FF, (**b**) AACI + FF, and (**c**) AWDT from the MWI plant. (The ID number and concentration of each VOC species is listed in Table S2).

Five alkanes/alkenes were identified in the flue gas from waste incineration, namely styrene, n-hexane, pentane, dodecene, and 1-decene. As shown in Figure 3a, the total concentration of these compounds increased significantly after treatment by the ACI + FF system, rising from 617.0 ± 100.3 μg/m^3^ to 2102.9 ± 293.2 μg/m^3^, corresponding to a negative removal efficiency of −240.8%. Styrene showed the most pronounced increase, from 528.4 ± 69.2 μg/m^3^ to 1890.5 ± 205.6 μg/m^3^. A similar phenomenon was reported by Setyan et al. [8]. This counterintuitive trend may be attributed to several interrelated mechanisms.

The operating temperature of the FF must be maintained above 130 °C to prevent hygroscopic salts (primarily calcium chloride) from absorbing moisture and agglomerating, which could lead to severe corrosion and clogging issues [20]. This temperature constraint also directly limits the use of activated carbon for VOCs. Since VOCs are primarily captured by activated carbon through physical adsorption, elevated temperatures significantly suppress its adsorption capacity. Ma et al. [21] investigated the effect of activated carbon injection temperature on the adsorption performance of toluene and chlorobenzene. Their results showed that when the temperature increased from 90 °C to 150 °C, the adsorption capacities for toluene and chlorobenzene decreased from 49.9 mg/g and 80.3 mg/g to 20.6 mg/g and 30.5 mg/g, respectively. Similar results were reported by Tsai et al. [22].

In this study, the working temperature of activated carbon ranged between 150 °C and 180 °C, which limited its effectiveness in capturing alkanes and alkenes. Furthermore, the fly ash and activated carbon captured in the baghouse filter form a packed bed, where variations in adsorption affinity among different VOC species may shift the adsorption–desorption equilibrium, resulting in the re-release of previously adsorbed compounds [23]. Treese et al. [24] found that when acetone, toluene, and cyclohexane coexisted, toluene preferentially adsorbed onto the activated carbon surface. Once the adsorption sites were saturated, weaker-adsorbing molecules were displaced by those with stronger affinity [25]. This desorption effect induced by competitive adsorption may be a key factor contributing to the increased concentrations of alkane/alkene. In addition, incinerator fly ash contains various metal oxides that may catalyze the formation of small-molecule alkanes and alkenes, further contributing to their elevated concentrations in the flue gas [26,27].

Notably, the subsequent WDT device exhibited excellent removal efficiency for alkanes and alkenes, achieving a removal rate of 97.32%, with the outlet concentration reduced to 56.3 ± 21.6 μg/m^3^. This high-efficiency removal can be attributed to several synergistic physicochemical mechanisms that occur within the wet scrubbing process.

First, the rapid reduction in flue gas temperature within the WDT promotes the condensation of high-boiling-point hydrocarbons. As the gas stream cools, these compounds transition from the vapor phase to liquid droplets, which are subsequently captured by the circulating scrubbing liquid.

Second, although alkanes and alkenes are generally hydrophobic, the intense gas–liquid contact, turbulent mixing, and the presence of surfactant-like constituents in the scrubbing slurry enhance their effective absorption. Fine mist droplets generated during atomization also increase the interfacial area, improving mass transfer and facilitating the dissolution or entrainment of low-polarity organic molecules.

Third, during the scrubbing process, VOCs may undergo physicochemical interactions with dissolved inorganic species (e.g., SO_4_^2−^, Cl^−^, Na^+^, and Ca^2+^). These interactions can promote the formation of condensable particulate matter (CPM) via homogeneous or heterogeneous nucleation pathways. Once converted into particulate-phase products or associated with growing droplets, these species are readily removed through sedimentation, impaction, or subsequent demisting stages [28,29].

Overall, the combined effects of thermal condensation, enhanced gas–liquid mass transfer, and chemical or nucleation-driven phase transformation collectively contribute to the exceptional removal efficiency observed for alkanes and alkenes in the WDT device.

### 3.3. Distribution Characteristics of O-VOCs

The distribution of O-VOCs was similar to that of alkanes/alkenes, as shown in Figure 3b. The ACI + FF system led to a significant increase in O-VOC concentrations in the flue gas—from 90.7 ± 44.8 μg/m^3^ to 417.6 ± 147.8 μg/m^3^—resulting in a negative removal efficiency of −360.4%. In contrast, the WDT device demonstrated excellent synergistic removal performance, reducing the concentration from 417.6 ± 147.8 μg/m^3^ to 52.4 ± 11.9 μg/m^3^, corresponding to a removal efficiency of 87.5%.

In general, non-polar molecules tend to adsorb more readily onto adsorbents lacking polar functional groups [30,31]. As a typical non-polar pollutant, dioxins are efficiently removed using activated carbon with a highly developed porous structure and non-polar surface chemistry. However, O-VOCs are generally polar, and this polarity weakens their adsorption capacity on activated carbon. As a result, in multi-component systems, O-VOCs are more susceptible to being displaced by other molecules during competitive adsorption, leading to desorption. This mechanism is likely the primary reason for the significant increase in O-VOC concentrations. Additionally, dioxins or other organic compounds previously adsorbed onto activated carbon or fly ash may undergo catalytic transformation into O-VOCs in the presence of metal oxides, which is also considered an important source of O-VOCs.

As for the scrubbing process, in addition to the condensation effect caused by the sharp drop in flue gas temperature, the high water solubility of many O-VOCs enables effective dissolution through gas–liquid contact with the scrubbing liquid [32]. Furthermore, O-VOCs may associate with particulate matter via gas–particle partitioning and can subsequently be captured by the WDT device.

### 3.4. Distribution Characteristics of Aromatics

The distribution behavior of aromatic hydrocarbons differed from that of alkanes/alkenes and O-VOCs, as shown in Figure 3. Both the ACI + FF system and the WDT device effectively reduced the concentration of aromatic hydrocarbons in the flue gas—from 303.4 ± 49.5 μg/m^3^ to 152.6 ± 47.9 μg/m^3^ and further to 39.5 ± 11.6 μg/m^3^—corresponding to removal efficiencies of 49.7% and 74.1%, respectively.

Compared to alkanes/alkenes and O-VOCs, aromatics exhibit strong π–π interactions with the activated carbon surface due to the conjugated π-electron system of the benzene ring [33,34]. This interaction enhances their affinity for physical adsorption within the microporous structure of activated carbon. Moreover, the bed layer formed by activated carbon and fly ash in the baghouse filter increases the residence time of flue gas, further improving the adsorption efficiency of aromatic compounds. Chen and He [35] also found that although rising adsorption temperatures reduce total adsorption capacity, the selectivity of activated carbon for toluene increases, while that for polar compounds like ethanol decreases significantly. This could explain why O-VOCs showed the highest proportion of secondary release after passing through the ACI + FF system.

As for the WDT device, although aromatics have relatively low water solubility, their high boiling points enhance condensation during the rapid cooling of the flue gas. In addition, microdroplets or emulsified particles of organic solvents in the scrubbing liquid may encapsulate and remove part of the aromatic hydrocarbons. Nonetheless, compared to alkanes/alkenes and oxygenated VOCs, the overall removal efficiency of aromatic hydrocarbons by the scrubber remains relatively lower.

### 3.5. Distribution Characteristics of Halogenated Hydrocarbons

The distribution characteristics of halogenated hydrocarbons were similar to those of aromatic hydrocarbons, as shown in Figure 3, exhibiting a continuous decreasing trend—from 491.5 ± 179.6 μg/m^3^ to 266.8 ± 100.8 μg/m^3^ and further to 189.9 ± 53.5 μg/m^3^—corresponding to removal efficiencies of 45.7% and 28.8%, respectively.

Although the total concentration of halogenated hydrocarbons shows a decreasing trend after passing through the ACI + FF system, the concentration changes in individual compounds are not uniform. Compounds such as vinyl chloride (398.1 ± 152.3 μg/m^3^ → 15.8 ± 4.4 μg/m^3^) and methyl bromide (19.6 ± 0.6 μg/m^3^ → 12.1 ± 3.1 μg/m^3^) exhibit significant reductions, mainly due to effective adsorption and removal by activated carbon and fly ash. Halogen atoms (e.g., Cl and Br) possess high electron density and are easily polarizable, which greatly increases the molecular polarizability [36]. Since van der Waals forces, particularly London dispersion forces, are positively correlated with polarizability, the adsorption affinity of these compounds is significantly enhanced [37]. In contrast, a few small-molecule halogenated hydrocarbons, such as bromoform (13.8 ± 2.0 μg/m^3^ → 22.1 ± 8.4 μg/m^3^), 1,2-dichloroethane (5.5 ± 2.0 μg/m^3^ → 27.6 ± 13.6 μg/m^3^), and dichloromethane (12.4 ± 4.8 μg/m^3^ → 41.9 ± 13.6 μg/m^3^), show an increasing concentration trend. This may be attributed to the catalytic degradation of trace amounts of dioxins by metal oxides present in fly ash, which can promote dechlorination, ring-opening, and subsequent recombination reactions, thereby generating halogenated organic byproducts. Similar catalytic decomposition pathways and the formation of chlorinated intermediates have been reported in previous studies investigating the transformation of dioxins and related aromatic pollutants on metal-oxide surfaces [38,39].

Due to low water solubility, halogenated hydrocarbons are not efficiently removed by scrubbing with water alone. Their reduction relies primarily on condensation driven by the drop in flue gas temperature. Moreover, based on the main organic components of CPM (alkanes, alkenes, lipids, and organosilicon compounds), halogenated hydrocarbons are generally not prone to nucleate via heterogeneous condensation mechanisms to form CPM [40,41,42,43]. This explains why the WDT device exhibits the poorest synergistic removal efficiency for halogenated hydrocarbons. Notably, the concentrations of certain small-molecule halogenated hydrocarbons even increase after passing through the WDT device, such as bromoform (22.1 ± 8.4 μg/m^3^ → 41.8 ± 6.4 μg/m^3^), chloroform (3.7 ± 2.4 μg/m^3^ → 15.7 ± 5.0 μg/m^3^), tetrachloroethylene (2.1 ± 0.5 μg/m^3^ → 20.6 ± 8.0 μg/m^3^), and trichloroethylene (1.3 ± 0.8 μg/m^3^ → 9.9 ± 3.7 μg/m^3^). This increase is likely due to secondary release caused by desorption from the desulfurization solution or from gypsum. Similar observations were reported by Sun et al. [15], who found that the concentrations of halogenated hydrocarbons and aromatics increased after flue gas passed through the FGD unit in coal-fired boilers. Mechanistically, Cheng et al. [44] showed that VOCs with higher aqueous solubility dominate the gas–liquid interface, suppressing uptake of low-solubility VOCs and provoking secondary desorption, which manifests as elevated outlet concentrations.

**Figure 4:**
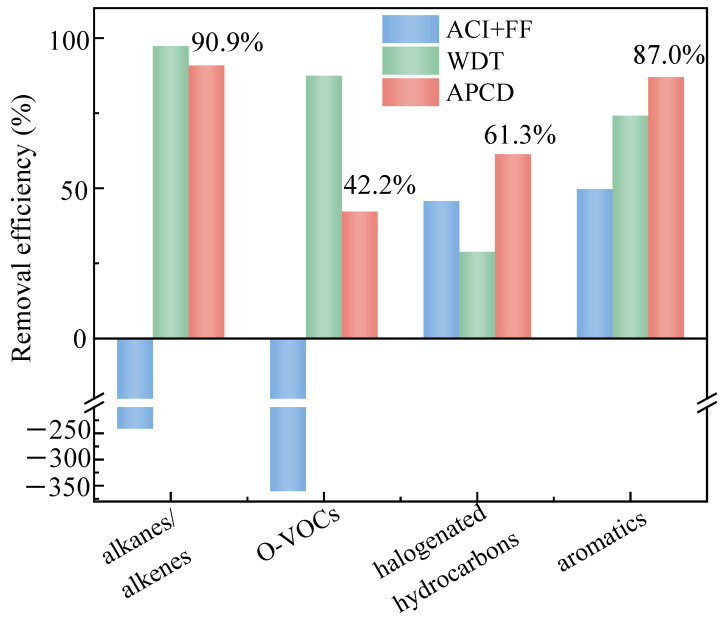
VOC removal performance of existing pollutant control equipment in MSWI plants.

In summary, the overall synergistic removal efficiencies of the existing APCDs for the four categories of VOCs are ranked as follows: alkanes/alkenes (90.9%) > aromatic hydrocarbons (87.0%) > halogenated hydrocarbons (61.3%) > O-VOCs (42.2%), with the TVOC removal efficiency of 77.5%, as shown in Figure 4. Table S3 presents a literature review on the synergistic VOC removal performance of APCD systems in coal-fired power plants. Overall, the APCD system at the MWI plant investigated in this study demonstrates comparable VOC removal performance to that of coal-fired power plants. The ACI + FF system primarily removes aromatic and halogenated hydrocarbons via physical adsorption, while the WDT device achieves more effective removal of alkanes/alkenes and O-VOCs through dissolution, condensation, and nucleation. The integration of these systems enables a stepwise, broad-spectrum purification of VOCs in the flue gas stream.

### 3.6. VOC Emission Characteristics in MWI Flue Gas

50 target VOCs were determined in the flue gas at the AWDT sampling location from the MWI plant, with a total concentration of 338.2 ± 98.1 μg/m^3^. The results indicate that 9 VOCs were completely removed after the flue gas passed through the ACI + FF system and WDT device. Table 3 summarizes reported VOC emission concentrations from MWI plants, showing that TVOC concentrations range from 16.8 μg/m^3^ to 4.3 mg/m^3^. The TVOC concentration measured in this study falls within this typical range.

The large variation in reported VOC concentrations can be primarily attributed to differences in the number of VOC species determined, sampling method, and the configuration of APCDs used in different plants. For instance, Setyan et al. [8] did not quantify halogenated hydrocarbons in the flue gas, resulting in a total VOC concentration of only 16.8–27.1 μg/m^3^, which is significantly lower than other studies [9,45]. In contrast, Sun et al. [11] and Chen et al. [10] used similar APCDs and determined a comparable number of VOC species. However, due to the differences in sampling methods, the concentrations of VOCs varied by as much as 6.7 times.

**Figure 5:**
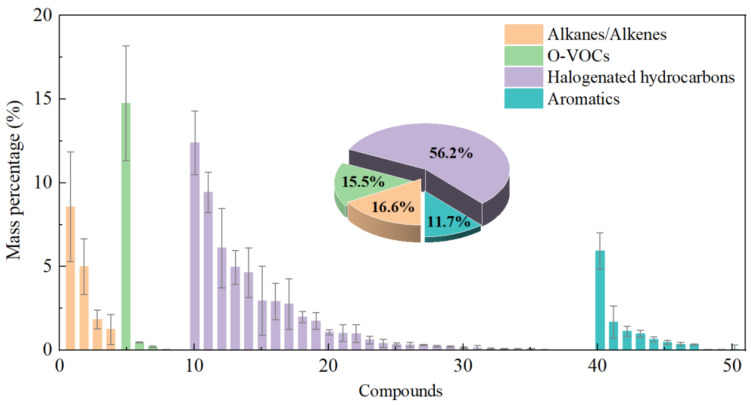
VOCs source profile of the emissions from the MWI plant (The ID number of each VOC species is listed in Table S4, Supplementary Material).

Despite the differences in TVOC concentrations, the compositional profiles of VOCs from various MWI plants exhibit strong similarities, typically comprising aromatic hydrocarbons, halogenated hydrocarbons, alkanes, and O-VOCs. Representative VOCs include tetrachloroethylene, benzene, and toluene. In this study, halogenated hydrocarbons were the dominant group, accounting for 56.2% of total VOCs, followed by alkanes/alkenes (16.6%), oxygenated VOCs (15.5%), and aromatic hydrocarbons (11.7%), as shown in Figure 5. Compared with literature data [9,10,11], the proportion of halogenated hydrocarbons observed here is notably higher, likely due to the specific composition of incinerated waste, such as a higher content of chlorine-containing plastics or electronic waste.

Among the four groups of VOCs, bromoform, acetone, n-hexane, and toluene were the representative compounds, accounting for 12.4%, 14.8%, 8.6%, and 5.9% of TVOCs, respectively. Acetone is the most abundant VOCs emitted into the atmosphere from the MWI plant, a finding that has also been reported in the study by Sun et al. [11]. The emissions of these substances warrant serious attention due to their potential health hazards. For example, bromoform has a strong inhibitory effect on the central nervous system. Inhalation of high concentrations can cause dizziness, vomiting, and even lead to liver and kidney damage [46]. Acetone, a widely used solvent, can cause respiratory tract irritation, headache, and drowsiness upon acute exposure, while chronic exposure may impair liver and kidney function [47]. n-Hexane is known for its significant neurotoxicity, and prolonged exposure can lead to peripheral neuropathy, characterized by numbness and muscle weakness in the limbs [48]. Toluene exerts its toxic effects primarily by suppressing the central nervous system, potentially causing dizziness and cognitive impairment, and it also has reproductive and developmental toxicity [49]. Although the International Agency for Research on Cancer (IARC) has not classified these substances as carcinogenic or possibly carcinogenic to humans, their toxicological effects have been confirmed in various animal studies and occupational exposure investigations [50,51]. Therefore, enhancing the control of emissions from waste incineration is of great importance for protecting public health.

To better estimate VOC emissions from MWI plants, the EF was calculated using Equation (1). In this study, the EF for MWI plant was determined to be (1.9 ± 0.6) × 10^3^ ng/g-waste. Table 3 compares the *EFs* with values reported in previous studies: Beylot et al. [52] reported an *EFs* of 4.68 × 10^3^ ng/g-waste for 90 French MWI plants, while Yan et al. [53] reported 5.24 × 10^3^ ng/g-waste for 12 MWI plants in Beijing. These values are of the same order of magnitude as this study, but are far below the emission factor (7.4 × 10^5^ ng/g-waste) recommended by the Technical Manual for Compiling Urban Air Pollutant Emission Inventories. This suggests that using outdated emission factors would significantly overestimate current VOC emissions from MWI plants, mainly due to the high coverage and improved efficiency of modern APCD systems.

Based on the calculated *EFs*, the annual TVOC emissions from the MWI plant in this study were estimated to be (1.9 ± 0.6) × 10^5^ g. In the absence of air pollution control devices such as ACI + FF system and WDT device, the *EFs* of TVOC emission increase to (8.4 ± 2.1) × 10^3^ ng/g-waste, corresponding to an annual TVOC emission of up to (8.4 ± 2.1) × 10^4^ g. This indicates that the current APCDs can reduce TVOC emissions by approximately 6.5 × 10^4^ g annually. Such a significant reduction highlights the critical role of multistage flue gas purification processes in controlling VOC emissions.

**Table 3:** VOCs characteristics and emission factor of MWI plant.

Region	Number of VOCs	VOCs Concentration (μg/m^3^)	Emission Factor (ng/g-Waste)	VOCs Characters	Typical Substance	Sampling Method	Ref.
Guangzhou Province	25	270.63 ± 2.84	-	aromatics (63.66%), halo hydrocarbons (11.49%), alkanes (16.17%), O–VOCs (3.19%)	Chlorobenzene, tetrachloroethylene	adsorption tube	[9]
Switzerland (Hinwil)	25	16.75	-	Alkanes (61.37%), aromatics (30.63%) O–VOCs (8.00%)	p-xylene, nonane, benzaldehyde	adsorption tube	[8]
Switzerland (Giubiasco)	25	20.75–27.08	-	alkanes (51.58%), aromatics (36.09%) O–VOCs (12.33%)	p-xylene, nonane, benzaldehyde		
Henan Province	89	4.28 mg/m^3^	16 × 10^3^	aromatics (38.4%), halo hydrocarbons (28.8%), O–VOCs (14.3%), alkanes (12.8%)	Tetrachloroethylene, Hexachloro-1,3-butadiene, Acetaldehyde	Tedlar bag, summa canister, adsorption tube	[10]
Shandong province	30	116.3 ± 15.7	0.7 × 10^3^ ± 0.1 × 10^3^	aromatics (28–47%), halo aromatics (22–52%), halo aliphatics (19–30%), Chennai (0.3–0.5%)	Ethylbenzene, m/p-xylene, tetrachloroethylene	Tedlar bag	[45]
North China plan	102	635.3 ± 588.8	2.43 × 10^3^ ± 2.27 × 10^3^	aromatics (62.1%), O–VOCs (16.0%), halo hydrocarbons (10.0%), alkanes (6.6%)	acetone, 1,2,4-trimethylbenzene, benzene	Tedlar bag	[11]
Beijing	-	-	5.24 × 10^3^ ± 0.15 × 10^3^	-			[53]
China	50	338.2 ± 98.0	1.89 × 10^3^ ± 0.55 × 10^3^	halo hydrocarbons (56.2%), aromatics (11.7%%), O–VOCs (15.5%), alkanes (16.6%)	Acetone, Bromobenzene, n-hexane, toluene	adsorption tube	This study

## 4. Conclusions

In this study, the VOC concentration of an MWI plant with APCD systems were measured at different sampling locations by a sorbent tubes sampling system.

A total of 59 VOC species were determined for the inlet of ACI device, with a total concentration of 1502.6 ± 374.2 μg/m^3^. Due to the high content of plastic packaging materials in the waste feed, styrene (528.4 ± 69.2 μg/m^3^) and vinyl chloride (393.1 ± 152.3 μg/m^3^) were identified as the dominant VOCs generated during incineration. After passing through the ACI + FF system and WDT device, the TVOC concentration significantly decreased to 338.2 ± 98.1 μg/m^3^, with 9 VOC species being nearly completely removed.

Following the APCD treatment, the dominant VOCs in the emitted flue gas were bromoform, acetone, n-hexane, and toluene. The adsorption capacity of ACI + FF system, combined with the condensation, scrubbing, and nucleation-promoting effects of the WDT device, played a critical role in VOC removal. The emission factor of VOCs was (8.4 ± 2.1) × 10^3^ ng/g-waste. Based on the APCDs’ synergistic removal performance, the MWI plant can reduce VOC emissions by approximately 6.5 × 10^4^ g annually. These findings highlight the importance of efficient APCD systems in waste incineration and provide essential data to support VOC inventory refinement and environmental policy development.

## Figures and Tables

**Table 1:** The main components of municipal waste.

Organics						Inorganics
Plastic/Rubber Products	Cotton fabric	Woodware	Food waste	Medicine	Paper products	Glassware/metalware/muck
22%	7%	6%	37%	4%	11%	13%

**Table 2:** Elemental and proximate analyses of municipal waste.

Materials	Elemental Analysis (Dry Basis, wt.%)					Proximate Analysis (wt.%)				HHV (MJ/kg)
	C	H	O	N	S	VM	MO	FC	Ash	
Municipal wastes	52.9	7.0	29.8	1.3	0.7	78.1	3.8	9.8	8.3	20.19 ± 0.68

VM: Volatile matter; MO: Moisture; FC: Fixed carbon; HHV: Higher heating value (MJ/kg).

## Data Availability

The original contributions presented in this study are included in the article/Supplementary Materials. Further inquiries can be directed to the corresponding author.

## Supplementary Materials

The following supporting information can be downloaded at: https://www.mdpi.com/article/10.3390/toxics13121067/s1, Table S1: VOCs species measured during chemical analysis; Table S2: Information on the concentrations of VOCs detected in the flue gas from the MWI plant; Table S3: APCDs removal efficiency of VOCs; Table S4: The sequence of VOCs in Figure 5.
