# Supplementary material for: Impact of Air Pollution Control Devices on VOC Profiles and Emissions from Municipal Waste Incineration Plant
Source: Toxics. 2025 Dec 11;13(12):1067. doi: 10.3390/toxics13121067 (PMC12737556; doi:10.3390/toxics13121067)
Supplement: Supplementary file 1 [file toxics-13-01067-s001.zip › toxics-3993415-supplementary.pdf]

Supplementary material

Impact of Air Pollution Control Devices on VOC Profiles and Emissions from Municipal Waste Incineration Plant

Jun Liu [1,2,*], Duanhe Zhao [1], Fei Wu [3], Huanhuan Luo [3], Daxiang Hou [1] and Yue Peng [2,4]

[1] *School of Petroleum and Natural Gas Engineering, Changzhou University, Changzhou 213164, China; s24040858026@smail.cczu.edu.cn (D.Z.); s24040858029@smail.cczu.edu.cn (D.H.)*

[2] *Key Laboratory of Oil-Gas & New-Energy Storage and Transportation Technology of Jiangsu Province Higher Education Institutes, Changzhou University, Changzhou 213164, China; pengyue@bipt.edu.cn*

[3] *Huaneng Hunan Yueyang Power Generation Co., Ltd., Yueyang 414000, China; wufei1503@163.com (F.W.); lh10172025@163.com (H.L.)*

[4] *Department of Environmental Engineering, Beijing Institute of Petrochemical Technology, Qingyuan North Road 19, Beijing 102617, China*

* Correspondence: lj0522@cczu.edu.cn

Number of Tables: 4

Table S1 VOCs species measured during chemical analysis

No.	Retention time (min)	Characteristic fragment ions (*m/z*)	CAS	Species of VOC
1	4.43	62	75-01-4	Vinyl chloride
2	4.45	94	74-83-9	Bromomethane
3	4.5	64	75-00-3	Chloroethane
4	4.79	58	67-64-1	Acetone
5	4.89	45	67-63-0	Isopropyl alcohol
6	4.91	101	75-69-4	Trichlorofluoromethane
7	5.02	61	75-35-4	1,1-Dichloroethene
8	5.2	49	75-09-2	Dichloromethane
9	5.62	61	156-60-5	trans-1,2-Dichloroethene
10	5.83	63	75-34-3	1,1-Dichloroethane
11	6.11	57	110-54-3	n-Hexane
12	6.32	61	156-59-2	cis-1,2-Dichloroethene
13	6.44	43	141-78-6	Ethylacetate
14	6.57	49	74-97-5	Bromochloromethane
15	6.67	83	67-66-3	Chloroform
16	7.37	62	106-93-4	1,2-Dichloroethane
17	7.58	78	71-43-2	Benzene
18	7.60	117	56-23-5	Carbon tetrachloride
19	8.59	57	96-22-0	3-Pentanone
20	8.68	130	79-01-6	Trichloroethene
21	8.75	63	107-04-0	1,2-dichloropropane
22	8.78	75	10061-01-5	cis-1,3-Dichloropropene
23	8.84	174	74-95-3	Dibromomethane
24	9.12	83	75-27-4	Bromodichloromethane
25	10.94	91	108-88-3	Toluene
26	12.19	129	124-48-1	Dibromochloromethane
27	12.29	166	127-18-4	Tetrachloroethene
28	12.52	107	106-93-4	1,2-Dibromoethane
29	12.54	43	123-86-4	n-Butyl acetate
30	13.73	112	108-90-7	Chlorobenzene
31	14.23	91	100-41-4	Ethylbenzene
32	14.31	43	108-65-6	Propylene Glycol Methyl Ether Acetate
33	14.52	91	108-38-3	m-Xylene
34	14.58	91	106-42-3	p-Xylene
35	15.12	43	110-43-0	2-Heptanone
36	15.26	104	100-42-5	Styrene
37	15.3	91	95-47-6	o-Xylene
38	15.33	173	75-25-2	Bromoform
39	16.07	108	100-66-3	Anisole
40	16.19	83	630-20-6	1,1,1,2-Tetrachloroethane
41	16.31	105	526-73-8	1,2,3-Trimethylbenzene
42	17.27	91	106-43-4	4-Chlorotoluene
43	17.46	91	103-65-1	Propylbenzene
44	18.34	41	872-05-9	1-Decene
45	18.46	119	98-06-6	tert-Butylbenzene
46	18.54	105	95-63-6	1,2,4-Trimethylbenzene
47	18.95	146	541-73-1	1,3-Dichlorobenzene
48	19.01	105	135-98-8	sec-Butylbenzene
49	19.23	146	106-46-7	1,4-Dichlorobenzene
50	19.45	119	99-87-6	p-Isopropyltoluene
51	19.78	146	95-50-1	1,2-Dichlorobenzene

52	20.35	91	104-51-8	n-Butylbenzene
53	21.18	58	821-55-6	2-Nonanone
54	21.37	157	107-06-2	1,2-Dibromo-3-chloropropane
55	23.60	180	120-82-1	1,2,4-Trichlorobenzene
56	23.66	69	112-41-4	1-Dodecene
57	23.85	128	91-20-3	Naphthalene
58	24.25	225	87-68-3	Hexachlorobutadiene
59	24.3	180	87-61-6	1,2,3-Trichlorobenzene

Note: Compounds in this table are listed in the order of their GC–MS retention times, corresponding to the analytical sequence used during VOC identification. This ordering facilitates traceability and comparison of chromatographic results.

Table S2 Information on the concentrations of VOCs detected in the flue gas from the MWI plant.

No.	Species of VOC	Concentration (μg/mg^3)		
		BACI+FF	AACI+FF	AWDT
		Alkanes/alkenes		
1	Styrene	528.38 ± 69.23	1890.50 ± 205.57	16.88 ± 5.61
2	n-Hexane	68.83 ± 22.94	170.09 ± 71.49	28.97 ± 11.04
3	Pentane	9.26 ± 3.09	33.75 ± 14.73	6.24 ± 1.92
4	1-Dodecene	5.74 ± 3.47	1.95 ± 0.22	-
5	1-Decene	4.81 ± 1.59	7.90 ± 1.18	4.22 ± 3.02
		O-VOCs		
6	Acetone	75.54 ± 33.50	363.34 ± 133.65	49.90 ± 11.58
7	n-Butyl acetate	7.31 ± 6.29	23.76 ± 2.71	0.72 ± 0.17
8	Ethyl acetate	6.55 ± 4.82	24.47 ± 7.65	1.56 ± 0.10
9	Isopropyl alcohol	0.60 ± 0.08	0.36 ± 0.15	0.14 ± 0.02
10	2-Heptanone	0.32 ± 0.07	0.26 ± 0.16	-
11	2-Nonanone	0.24 ± 0.01	0.67 ± 0.28	-
12	Propylene Glycol Methyl Ether Acetate	0.13 ± 0.07	4.67 ± 3.16	0.08 ± 0.07
13	Anisole	-	0.13 ± 0.02	-
		Aromatics		
14	Benzene	160.00 ± 14.24	11.94 ± 5.09	5.68 ± 3.22
15	Naphthalene	32.00 ± 7.42	10.99 ± 1.90	1.57 ± 0.40
16	1,2,4-Trimethylbenzene	28.70 ± 3.33	18.43 ± 8.54	3.29 ± 0.71
17	Toluene	28.10 ± 12.00	48.20 ± 9.20	20.06 ± 3.68
18	m-Xylene	19.39 ± 5.13	27.50 ± 5.32	2.20 ± 0.52

19	o-Xylene	11.35 ± 2.39	12.32 ± 6.21	3.86 ± 1.03
20	p-Xylene	7.29 ± 1.32	7.99 ± 4.19	1.21 ± 0.34
21	Ethylbenzene	5.23 ± 1.52	10.49 ± 5.61	1.18 ± 0.15
22	sec-Butylbenzene	3.82 ± 1.21	0.80 ± 0.53	-
23	tert-Butylbenzene	2.68 ± 0.09	0.46 ± 0.57	-
24	p-Isopropyltoluene	1.89 ± 0.32	1.59 ± 0.64	0.16 ± 0.02
25	n-Butylbenzene	1.68 ± 0.48	0.84 ± 0.28	0.13 ± 0.96
26	Propylbenzene	1.28 ± 0.04	1.05 ± 0.33	0.17 ± 0.02
27	1,2,3-Trimethylbenzene	0.41 ± 0.03	0.55 ± 0.29	0.07 ± 0.02
		Halogenated hydrocarbons		
28	Vinyl chloride	393.07 ± 152.26	15.81 ± 4.36	0.73 ± 0.10
29	trans-1,2-Dichloroethene	26.27 ± 12.28	24.36 ± 9.05	1.11 ± 0.46
30	Bromomethane	19.59 ± 0.65	12.09 ± 3.14	3.64 ± 0.51
31	Bromoform	13.76 ± 2.04	22.10 ± 8.42	41.88 ± 6.41
32	1,2-Dichloroethane	12.38 ± 4.83	41.91 ± 13.55	9.35 ± 5.05
33	Dichloromethane	5.49 ± 1.98	27.64 ± 18.53	10.01 ± 6.98
34	1,4-Dichlorobenzene	4.65 ± 0.09	4.05 ± 1.27	1.08 ± 0.13
35	1,2-Dichloropropane	2.98 ± 1.84	11.07 ± 5.21	3.37 ± 1.80
36	4-Chlorotoluene	2.07 ± 0.13	1.52 ± 0.74	0.25 ± 0.18
37	Chloroform	1.99 ± 0.75	3.70 ± 2.41	15.71 ± 5.00
38	Dibromomethane	1.85 ± 0.64	0.21 ± 0.13	0.83 ± 0.16
39	Hexachlorobutadiene	1.14 ± 0.08	0.71 ± 0.29	0.06 ± 0.02
40	Tetrachloroethene	1.05 ± 0.82	2.12 ± 0.47	20.64 ± 8.01
41	Carbon tetrachloride	1.05 ± 0.56	0.46 ± 0.30	1.15 ± 0.37
42	Bromochloromethane	1.03 ± 0.03	0.40 ± 0.24	1.41 ± 0.82

43	Bromodichloromethane	0.99 ± 0.19	0.71 ± 0.56	16.78 ± 3.41
44	Trichloroethene	0.60 ± 0.17	1.32 ± 0.80	9.90 ± 3.68
45	Chlorobenzene	0.35 ± 0.01	0.63 ± 0.10	2.09 ± 0.77
46	1,2,4-Trichlorobenzene	0.32 ± 0.15	2.34 ± 1.36	0.15 ± 0.02
47	1,2-Dichlorobenzene	0.23 ± 0.03	1.46 ± 0.52	0.46 ± 0.28
48	1,3-Dichlorobenzene	0.23 ± 0.01	0.59 ± 0.27	0.30 ± 0.14
49	Chloroethane	-	67.77 ± 26.91	5.91 ± 1.67
50	Trichlorofluoromethane	-	1.26 ± 0.93	-
51	1,1-Dichloroethene	-	0.61 ± 0.23	0.49 ± 0.16
52	1,1-Dichloroethane	-	0.31 ± 0.17	0.09 ± 0.01
53	cis-1,2-Dichloroethene	-	0.04 ± 0.03	0.28 ± 0.04
54	cis-1,3-Dichloropropene	-	12.73 ± 7.12	3.46 ± 1.70
55	Dibromochloromethane	-	2.10 ± 0.82	31.86 ± 4.08
56	1,2-Dibromoethane	-	2.08 ± 0.49	6.71 ± 1.12
57	1,2-Dibromo-3-chloropropane	-	1.77 ± 0.39	-
58	1,2,3-Trichlorobenzene	-	2.37 ± 1.68	0.22 ± 0.19
59	1,1,1,2-Tetrachloroethane	-	0.65 ± 0.54	-

Note: Compounds in this table are arranged in descending order of their concentrations measured at the ACI + FF inlet, highlighting the main types of VOCs released from municipal waste incineration. “-” indicates that the substance was either not detected or was present at a concentration below the detection limit.

Table S3 APCDs removal efficiency of VOCs

Fuel type	Boiler type	APCDs	Removal rate (%)	Ref.
Bituminous	Pulverized Coal Boiler	SCR+ESP+WFGD	87.6	[1]
Bituminous	Pulverized Coal Boiler	SCR+ LLT-ESP +WFGD	87.5	[1]
Bituminous	Pulverized Coal Boiler	SCR+LLT-ESP+WFGD+WESP	55.8	[2]
municipal waste	rotary kiln	BACI+FF+WDT	77.6	This study

Table S4 The sequence of VOCs in Fig. 5

Number	Species of VOC	Number	Species of VOC
1	n-Hexane	26	trans-1,2-Dichloroethene
2	Styrene	27	1,4-Dichlorobenzene
3	3-Pentanone	28	Dibromomethane
4	1-Decene	29	Vinyl chloride
5	Acetone	30	1,1-Dichloroethene
6	Ethylacetate	31	1,2-Dichlorobenzene
7	n-Butyl acetate	32	1,3-Dichlorobenzene
8	Isopropyl alcohol	33	cis-1,2-Dichloroethene
9	Propylene Glycol Methyl Ether Acetate	34	4-Chlorotoluene
10	Bromoform	35	1,2,3-Trichlorobenzene
11	Dibromochloromethane	36	1,2,4-Trichlorobenzene
12	Tetrachloroethene	37	1,1-Dichloroethane
13	Bromodichloromethane	38	Hexachlorobutadiene
14	Chloroform	39	1,2-Dibromo-3-chloropropane
15	Dichloromethane	40	Toluene
16	Trichloroethene	41	Benzene
17	1,2-Dichloroethane	42	o-Xylene
18	1,2-Dibromoethane	43	1,2,4-Trimethylbenzene
19	Chloroethane	44	m-Xylene
20	Bromomethane	45	Naphthalene
21	1,2-Dichloropropane	46	p-Xylene
22	cis-1,3-Dichloropropene	47	Ethylbenzene
23	Chlorobenzene	48	Propylbenzene
24	Bromochloromethane	49	p-Isopropyltoluene
25	Carbon tetrachloride	50	n-Butylbenzene

Note: Compounds in this table are arranged in descending order of their concentrations measured at the WDT outlet, highlighting the main substances that contribute most to the emissions. VOCs appearing in Table S1 but not here had concentrations below limit of quantification or were not detected at the WDT outlet.

References

[1] Liu J, Wang J, Cheng J, Zhang Y, Wang T, Pan W-P. Distribution and emission of speciated volatile organic compounds from a coal-fired power plant with ultra-low emission technologies. J Clean Prod. 2020;264:121686. DOI: https://doi.org/10.1016/j.jclepro.2020.121686.

[2] Cheng J, Liu J, Wang T, Sui Z, Zhang Y, Pan W-P. Reductions in Volatile Organic Compound Emissions from Coal-Fired Power Plants by Combining Air Pollution Control Devices and Modified Fly Ash. Energy Fuels. 2019;33:2926-33. DOI: https://doi.org/10.1021/acs.energyfuels.8b04277.
